# Critical Model Insight into Broadband Dielectric Properties of Neopentyl Glycol (NPG)

**DOI:** 10.3390/ma17164144

**Published:** 2024-08-21

**Authors:** Aleksandra Drozd-Rzoska, Jakub Kalabiński, Sylwester J. Rzoska

**Affiliations:** Institute of High Pressure Physics Polish Academy of Sciences, Sokołowska 29/37, 01-142 Warsaw, Poland

**Keywords:** neopentyl glycol, plastic crystals, orientationally disordered crystals, broadband dielectric spectroscopy, electric conductivity, dielectric constant, low-frequency changes in dielectric permittivity, discontinuous phase transitions, glassy dynamics

## Abstract

This report presents the low-frequency (LF), static, and dynamic dielectric properties of neopentyl glycol (NPG), an orientationally disordered crystal (ODIC)-forming material important for the barocaloric effect applications. High-resolution tests were carried out for 173K<T<440K, in liquid, ODIC, and solid crystal phases. The support of the innovative distortion-sensitive analysis revealed a set of novel characterizations important for NPG and any ODIC-forming material. First, the dielectric constant in the liquid and ODIC phase follows the Mossotti Catastrophe-like pattern, linked to the Clausius–Mossotti local field. It challenges the heuristic paradigm forbidding such behavior for dipolar liquid dielectrics. For DC electric conductivity, the prevalence of the ‘critical and activated’ scaling relation is evidenced. It indicates that commonly applied VFT scaling might have only an effective parameterization meaning. The discussion of dielectric behavior in the low-frequency (LF) domain is worth stressing. It is significant for applications but hardly discussed due to the cognitive gap, making an analysis puzzling. For the contribution to the real part of dielectric permittivity in the LF domain, associated with translational processes, exponential changes in the liquid phase and hyperbolic changes in the ODIC phase are evidenced. The novelty also constitutes tgδ temperature dependence, related to energy dissipation. The results presented also reveal the strong postfreezing/pre-melting-type effects on the solid crystal side of the strongly discontinuous ODIC–solid crystal transition. So far, such a phenomenon has been observed only for the liquid–solid crystal melting transition. The discussion of a possible universal picture of the behavior in the liquid phase of liquid crystalline materials and in the liquid and ODIC phases of NPG is particularly worth stressing.

## 1. Introduction

When a liquid is cooled, the discontinuous phase transition to a solid crystalline phase is the canonical phase sequence [1,2,3]. However, there are materials in which an additional mesophase can appear. A classic example is rod-like liquid crystalline (LC) molecular materials, where solely the orientational order can freeze, but the complete or ‘weakly’ limited translational disorder (freedom) remains. The isotropic liquid (I)–nematic (N) transition is a basic example [4,5,6,7,8]. There are also mesophasic materials, where the translational ‘freezing’ occurs in the crystalline lattice, but the orientational disorder (freedom) remains [9,10,11,12,13]. It appears for the orientationally disordered crystal (ODIC) mesophase, from the family of plastic crystals [11,12,13,14,15,16,17,18,19]. In LC- and ODIC-forming materials, upon further cooling, the discontinuous melting/freezing-type transition from the mesophase to the solid crystal phase can occur [7,9,20]. For ODIC-forming materials, such transition is often bypassed, and the solidification in the solid amorphous glass state can also occur for any experimental cooling rate [14,15,16,17,18,19,20].

It is claimed that ODIC mesophase appears for materials composed of globular or pseudo-globular molecules, where the free orientation of the molecules seems to be an inherent feature [12,13,16,19]. Nevertheless, plastic crystal mesophases are often also observed for molecules with a topology/shape that allows free rotation about any axis of their symmetry [14,15,16,17,18,19,20]. For molecules with an elongated, rod-like shape, a rotor/rotatory plastic crystal phase, where the ‘free’ rotations are associated with the dominant long axis of the molecule, can appear [21,22,23,24,25]. For disc-like molecules, a ‘free’ rotation related to the axis that is approximately perpendicular to the molecular surface can lead to the formation of ODIC mesophase. An example with an extensive research record is cyclooctanol and related compounds or their mixtures [16,17,18,20].

Worth stressing is the significance of the ODIC phase for the pre-vitreous dynamics studies on approaching the glass temperature ($T_{g}$), indicating the transition to the solid ‘orientationally amorphous’ (glass) state [14,15,16,17,18,19,20]. The phenomenal hallmark is the non-Arrhenius slowing down of the primary relaxation time [26,27,28,29,30], related to orientations of the permanent dipole moment coupled to molecules.

Neopentyl glycol (NPG) is the ODIC-forming compound of particular technological significance. It is the basis for resinous coatings, a component of lubricants and greases. It is used in the textile industry and is vital in the pharmaceutical and food industries [31,32]. The global market for NPG is worth ~ USD 2.5 billion in 2024 [32].

Recently, it has been shown that NPG can be a ‘breakthrough’ material for the new generation of coolers or air-conditioners based on the barocaloric effect of innovative implementations [33,34,35,36,37,38,39]. The existence of ODIC–crystal discontinuous phase transition at near room temperature in NPG has been known for a long time. Four years ago, it was shown that it is associated with a colossal entropy change: ΔS=300−500 J/Kkg, increasing with compressing [33,34]. This value is even higher than for omnipresent devices exploring vapor–liquid transition [33,34,35,36]. However, innovative NPG-based devices may have qualitative advantages, as follow: (i) minimal pollution threads for the environment, (ii) no impact on global warming, (iii) lesser usage of energy than for existing refrigeration technologies, and (iv) the possibility of ‘cool’ storage, with virtually no energy consumption.

Extensive experimental and modeling studies have been carried out for NPG and related systems, especially since 2019 [33,34,35,36,37,38,39]. Surprisingly, the discussion regarding dielectric properties is limited, although broadband dielectric spectroscopy (BDS) is the essential research method for ODIC-forming materials. In 1997, Tamarit et al. [40] presented the evolution of the primary relaxation time in ODIC phase of NPG for temperatures from ~353 K to 305 K, i.e., covering ca. 50% of the ODIC phase range. The slightly nonlinear changes in the Arrhenius scale plot, $\ln\tau \left(T\right)$ vs. 1/T, reveals the Super Arrhenius (SA) dynamics, considered the universalistic feature for pre-vitreous dynamics. In subsequent reports, the parameterization via the Vogel–Fulcher–Tamman (VFT) dependence, i.e., the replacement equation for the general SA relation, was shown as [41,42]:

(1) $$\tau \left(T\right)=\tau_{\infty }exp\left(\frac{E_{a}\left(T\right)}{RT}\right)\;\;\Rightarrow \tau \left(T\right)=\tau_{\infty }exp\left[\frac{D_{T}T_{0}}{T-T_{0}}\right]$$

where the left part is for the general SA equation, with the apparent (temperature-dependent) activation energy, $E_{a}\left(T\right).$ It reduces to the basic Arrhenius pattern for $E_{a}\left(T\right)=E_{a}=const$. The right part is for the VFT replacement equation. Equation (1) is for the supercooled liquid-like temperature domain, $T>T_{g}$, where $T_{0}<T_{g}$ is the extrapolated VFT singular temperature and $T_{g}$ is the glass temperature, which can be estimated via the empirical condition: *τ*$\left(T_{g}\right)=100s$. $D_{T}$ is the fragility strength parameter, and $D_{T}T_{0}=const$ [26,27].

The VFT equation is the commonly used dependence for describing the pre-vitreous dynamics, including the vitrifying ODIC phase. Notwithstanding, starting from the year 2006, the prevalence of the critical-like parameterization in the ODIC phase was evidenced [14,15,16,17,18]:

(2) $$\tau \left(T\right)=\tau_{0}{\left(T-T_{C}\right)}^{-\varphi }$$

where $T_{C}<T_{g}$ is the extrapolated singular temperature, and the exponent φ=9−15 for different ODIC-forming materials.

It is notable that the primary relaxation-time-focused BDS studies in NPG constitute a particular experimental challenge since they require multi-GHz-range measurements carried out in a relatively volatile and sensitive contaminated material.

The currently most-often-recalled report for BDS studies in NPG was published in 2021 [43]. It was related to frequencies, f≤1 MHz, and covered liquid, ODIC (called Phase I), and crystal (called Phase II) phases in the temperature range from 416 K to 293 K. The report focused on the evolution of DC electric conductivity, for which the portrayal via the parallel of VFT Equation (1) was shown [43]:

(3) $$\sigma \left(T\right)=\frac{A}{\sqrt{T}}exp\left(\frac{-B}{T-T_{V}}\right)$$

where A,B=const, and $T_{V}$ is the extrapolated singular temperature.

In [43], the description via the above relation was evidenced in the liquid phase for the range covering ~10 K and in the ODIC phase (denoted as Phase I) in the domain covering ~60 K, namely, starting 22 K below the melting temperature and terminating ~7 K before the transition to the low-temperature Phase II. For the latter, the basic Arrhenius dynamics ($E_{a}^{\sigma }=const$) is reported. In [43], also, spectra of imaginary parts of dielectric modulus, $M^{\prime \prime }\left(f\right)$, and electric impedance, $Z^{\prime \prime }\left(f\right)$, for four temperatures were superposed and discussed. The authors of [43] concluded: ‘… *In the plastic crystalline phase, the proton hopping mechanism is most likely the underlying ion-conducting mechanism because of the rotational disorder and intrinsic defects (vacancies…) of the NPG molecules. In the ordered crystalline phase, the proton conduction is presumed to follow the proton hopping mechanism as determined from the localized relaxation and the temperature dependence of* $\sigma_{DC}$ *(Arrhenius behavior)*’.

The in-depth insight into dielectric properties is essential for ODIC-forming materials since their properties are shaped by more-or-less freely rotating molecules translationally frozen in a crystalline network. The meaning of dielectric insight is strengthened by the coupling of rotating molecules to permanent dipole moments. For NPG, such essential evidence is surprisingly limited, in fact, to [40,41,43]. These reports generally focus on the plastic ODIC phase, suggesting the VFT portrayal. It is an ‘extremely flexible’ functional portrayal often used to describe dynamics in glass-forming systems.

Nevertheless, its fundamental significance can be questioned, and it should be considered rather as an effective ‘tool’. It is particularly evident for symmetry-limited glass formers, to which ODIC-forming materials belong. There is also temperature-limited evidence for dielectric constants, but only in the ODIC-phase-restricted temperature range, which can be questioned, as shown below. All these indicate a grand cognitive gap for basic NPG properties, a material valuable for significant innovative devices.

This report aims to fill the cognitive gap regarding NPG dielectric properties. Below, the results of high-resolution BDS studies in NPG for an extreme temperature range (173 K<T<440 K), i.e., covering all phases of NPG, for frequencies up to f<10 MHz are presented. Tests and analysis are focused on the static and low-frequency (LF) domains. We emphasize the latter since scaling relations describing this domain remain a cognitive puzzle. The presented results include the distortion-sensitive insight into electric conductivity behavior, revealing new scaling patterns in liquid and ODIC phases. Temperature changes in the dielectric constant indicate the Mossotti Catastrophe behavior, generally considered as ‘forbidden’ for liquid polar dielectrics. The evidence of such behavior covers the liquid phase and the ODIC mesophase, with the orientational freedom of permanent dipole moments.

## 2. Materials and Methods

**Figure 1:**
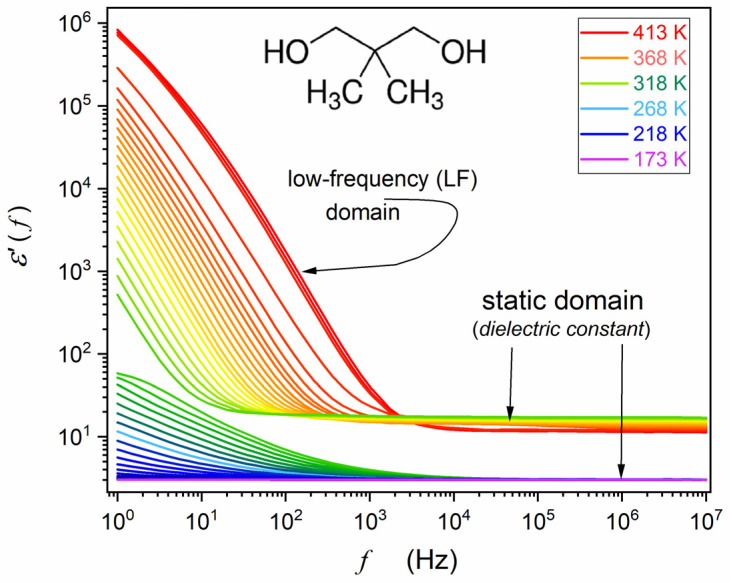
Frequency scans of real ($\varepsilon^{\prime }\left(f\right)$) contributions to dielectric permittivity in NPG. The characteristic temperatures are recalled in the figure for the orientation in the tested range. The molecular structure and relevant frequency domains are also shown. The static domain is for the horizontal region of the real part of dielectric permittivity, where a frequency shift does not change the value of $\varepsilon^{\prime }\left(f\right)$. Below the static domain is the low-frequency (LF) domain.

Studies were carried out in neopentyl glycol, i.e., 2,2-dimethyl-1,3-propanediol, presented also as $C_{5}H_{12}O_{2}$ or ${\left(CH_{3}\right)}_{2}C{\left(CH_{2}OH\right)}_{2}$). The structure is shown schematically in Figure 1. The compound was purchased from Sigma Company and used as delivered. Broadband dielectric spectroscopy (BDS) studies were conducted using the Novocontrol Alpha Analyzer in the frequency range from 1 Hz to 10 MHz, with U=1 V of the measuring field. It enabled 5–6-digit resolution. Samples were placed in the flat-parallel capacitor, made from gold-coated Invar, with a quartz ring as the spacer. The latter enabled the observation of the filling, which is significant in avoiding gas bubbles. The gap between plates was equal to 0.3 mm. The gap was supported by the quartz ring, as shown in [44], so it did not impact the measurement area between the capacitor plates. Such a design also enabled avoiding gas bubbles that can bias the results. The capacitor plated was made from gold-coated Invar. Generally, for the Alpha Analyzer, one can use voltages from 0.1 V to 40 V, but the optimal resolution reaching even 6 significant digits is related to U=1 V. It was possible to use such voltage in the given experiment due to the macroscale gap between capacitor plates, d=0.3 mm, with diameter 2r=20 mm, yielding E≈33 V/cm. For comparison, for micrometric gaps often used in dielectric studies, the intensity is essentially higher, namely, for d=10 μm and the lowest possible voltage U=0.1 V, one obtains E=1000 V/cm. Such intensities are in the domain of nonlinear dielectric effects, and the question arises of the biased impacts of gas bubbles or dust parasitic impurities. With such weak intensities of the measurement electric field, as applied in the given report, no influence on the dielectric constant could be detected despite the extreme sensitivity and resolution of the Alpha Analyzer.

**Figure 2:**
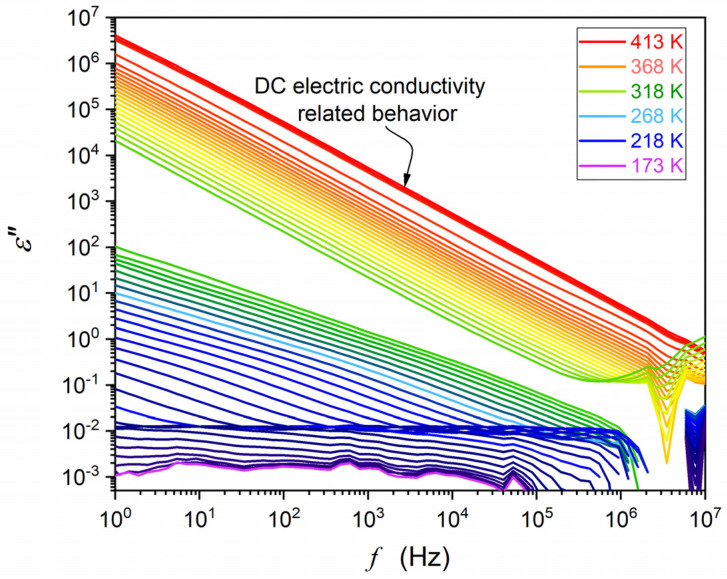
Frequency scans of the imaginary $\varepsilon^{\prime \prime }\left(f\right)$ to dielectric permittivity in NPG. The molecular structure and relevant frequency domains are also shown. The characteristic domains of the spectrum are indicated. The low-frequency part of $\varepsilon^{\prime \prime }\left(f\right)$ is related to DC electric conductivity: $\sigma =\sigma \left(DC\right)=\varepsilon_{0}\omega \varepsilon^{\prime \prime }\left(f\right)$, ω=2*πf*. Note that the DC electric conductivity exists in the liquid and ODIC phases, but it is absent in the solid crystal phase.

**Figure 3:**
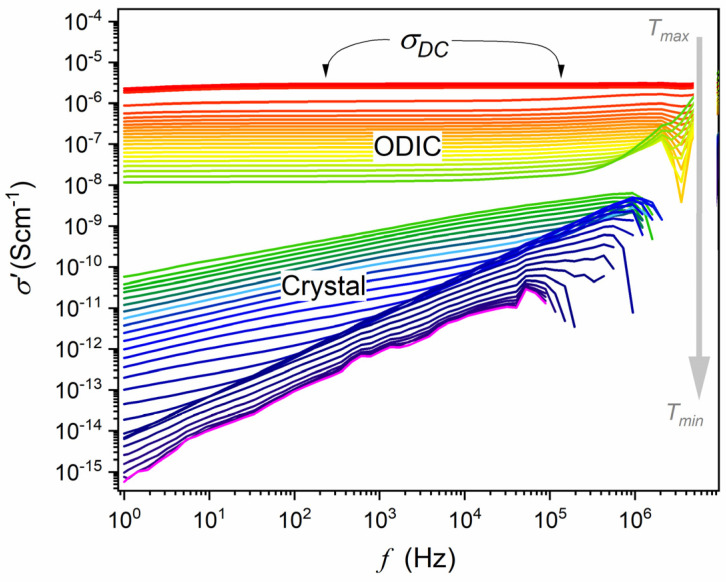
Frequency-related behavior of the real part of electric conductivity in the liquid, ODIC (Phase I), and crystal (Phase II) phases, based on data presented in Figure 2, namely, $\sigma^{\prime }=\varepsilon_{0}\omega \varepsilon^{\prime \prime }\left(f\right)$, where ω=2πf. Note that the DC electric conductivity is related to the horizontal behavior, which is absent in the solid crystal phase.

First, the capacitor was heated and filled with liquid NPG, which guaranteed optimal contact between the sample and the electrodes when cooling to subsequent phases. Temperatures ranging from 173 K to 440 K were tested, with the support of a Novoconrol Quattro thermostatic system yielding the control from 0.02 K to 0.1 K, depending on the temperature range. Figure 1 and Figure 2 show obtained frequency-related spectra for the sequence of tested temperatures presented as the complex dielectric permittivity, $\varepsilon^{*}=\varepsilon^{\prime }+{i\varepsilon }^{\prime \prime }$, and transformed to the complex electric conductivity representation. Obtained spectra are presented in Figure 1, Figure 2 and Figure 3. Distortions at the frequency limit were related to borders of the allowed impedance measurement for the spectrometer. The derivative analysis of data was supported by the subsequent numerical filtering using the Savitzky–Golay principle [45]. This report focuses on broadband dielectric properties of ODIC-forming NPG, which are essential because of the origins of the ODIC phase. As noted in the Introduction Section, they are surprisingly limited for NPG. Extensive material characterization regarding structural insight (XRD), Raman spectroscopy, DSC/DTA, etc., is presented in [33,34,38,39]. We did not explore these results in this report since they are insignificant to the presented reasoning. In the figures presented below, subsequent tested phases are additionally noted as ‘Phase I’ and ‘Phase II’ to support correlation with the results presented in [43].

## 3. Results and Discussion

### 3.1. Obtained BDS Spectra in NPG

Figure 1 and Figure 2 show the results of BDS measurements presented as the complex dielectric permittivity frequency scans for subsequent temperatures. They are indicated in colors, changing from red in the liquid state to orange–green in ODIC phase and blue–violet in the solid crystal phase. Gaps related to liquid–ODIC and ODIC–crystal phase transitions are also visible. In Figure 1, the structure of neopentyl glycol is also presented. Significant domains of spectra are indicated in Figure 1 and Figure 2. Spectra are split into three parts, related to the liquid, ODIC, and crystal phases, from top to bottom, with ‘gaps’ related to liquid–ODIC and ODIC–crystal phase transitions.

Other representations of electric impedance output detected in BDS studies can be convenient for some systems. For systems with the dominant impact of translational processes, the complex conductivity presentation, $\sigma^{*}=\sigma^{\prime }+i\sigma^{\prime \prime }$, offers a better insight. Notable is the link [46,47]:

(4) $$\sigma^{\prime }\left(f\right)=\omega \varepsilon_{0}\varepsilon^{\prime \prime }\left(f\right),\;\sigma^{\prime \prime }=\omega \varepsilon_{0}\left[\varepsilon^{\prime }-\varepsilon_{\infty }\right]$$

where $\varepsilon_{\infty }$ is for the infinite frequency, where only the atomic and electronic polarization contribute to the real part of dielectric permittivity, i.e., the contribution from permanent dipole moments dominates in the static domain, and the contribution from translational processes (LF domain) is absent. The circular frequency ω=2πf, and $\varepsilon_{0}\approx 8.854pFm^{-1}$ is the vacuum (free space) permittivity.

This is the case of NPG in the tested frequency range, as visible for $\varepsilon^{\prime \prime }\left(f\right)$ in Figure 2. Figure 3 shows spectra for the real part of electric conductivity, obtained via data presented in Figure 2, transformed via Equation (4). The horizontal part in Figure 3 defines the DC electric conductivity domain: $\sigma^{\prime }=\sigma_{DC}=\sigma$. Notably, the DC electric conductivity domain appears only in the liquid and ODIC phases, and there is no such behavior in the solid crystal phase, which shows the non-horizontal pattern of changes.

### 3.2. The Temperature Evolution of Dielectric Constant—Basic Reference

The dielectric constant is a fundamental characterization of dielectric properties of materials, introduced already by Michael Faraday [48]. This discovery and subsequent pioneering works by Ottaviano Mossotti [49] and Rudolf Clausius [50] led to the formation of Dielectric Physics and related material engineering topics.

**Figure 4:**
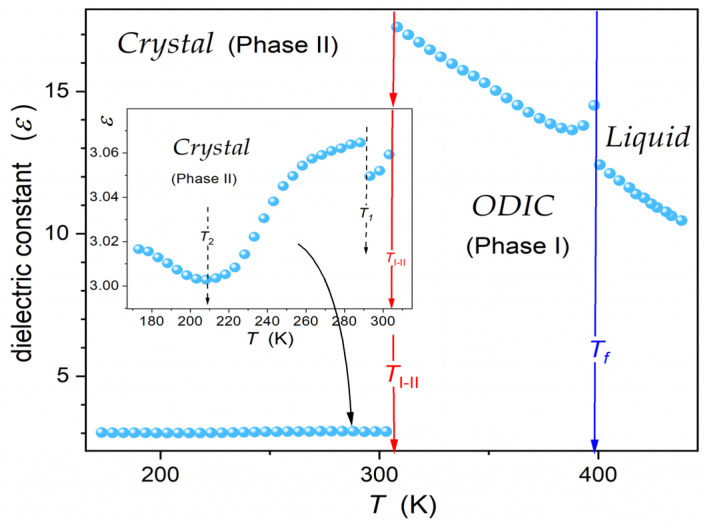
Temperature changes in the dielectric constant in subsequent phases of NPG. The arrows indicate the solidification to the plastic crystal phase at $T_{f}=399.5\;K$ and the orientationally disordered crystal (*ODIC*)–solid crystal (*Cr.*) transition at $T_{Cr-PCr}=307.75\;K$. The inset focuses on the solid crystal phase, showing hallmarks of ‘hidden’ transitions at $T_{1}=342.4\;K$ and $T_{2}=232.5\;K$. Such evidence was possible due to extreme resolution in BDS measurements.

Figure 4 presents the dielectric constant changes in NPG, covering the liquid, ODIC, and solid crystal phases down to 173 K. The eye inspection of temperature evolution in the central part of Figure 4 can suggest linear changes in $\varepsilon \left(T\right)$, except for ‘weak’ pre-melting/post-freezing-type effects in the ODIC phase, just below the transition to the liquid phase. Passing Liquid−ODIC and ODIC−Crystal is manifested by step-type changes in the dielectric constant: (i) for the liquid–ODIC transition, Δε≈2.16 or Δε≈0.73, if the impact of the pre-melting effect is omitted, and (ii) for the ODIC−Crystal transition, Δε≈14.3. Notably, the dielectric constant value, ε≈3.06, at the onset of the crystal phase agrees with those noted for crystalline dielectric materials with frozen translational and orientational freedom [46].

Timmermans, in his classic report [9], indicated a small value of the entropy change for the liquid–plastic crystal mesophase transitions, usually $\Delta S<20\;JK^{-1}{mol}^{-1}$, as its characteristic feature. It is ca. 10× less than that typically noted for the liquid–crystal transition [2,5]. It is notable that a similar ratio takes place when comparing Δε changes for Liquid−ODIC and ODIC−Crystal transition in NPG. Worth recalling is the recent report that discussed different contributions to entropy changes for a discontinuous melting transition [51]:

(5) $$\Delta S=\Delta s_{1}+\Delta s_{2}+\Delta s_{3}=\int_{0}^{T}\frac{C_{P,E}}{T}dT-\int_{P_{ref.}}^{P}v\alpha dP+\varepsilon_{0}\int_{0}^{E}v{\left(\frac{d\varepsilon }{dE}\right)}_{P}dE$$

The term $\Delta s_{1}$ is associated with internal energy, the term $\Delta s_{2}$ is related to compressibility, and $\Delta s_{3}$ is related to dielectric constant changes. One can expect that $\Delta s_{2}$ and $\Delta s_{3}$ can be particularly important for ODIC-forming materials. They refer to the ‘softness’ of the ODIC mesophase and the large ‘jump’ of Δε.

Notably, a similar sequence of ΔS values can be concluded for isotropic liquid–LC mesophase and LC mesophase–crystal transitions [5]. First, it is related to the weakly discontinuous phase transition behavior associated with critical-like, pre-transitional effects in the liquid phase [4,5,8,52]. Such a phenomenon has recently been shown for ODIC-forming cyclooctanol, based on the dielectric constant, nonlinear dielectric effect, and Kerr effect investigations [20]. Finally, the model for the common description of the liquid–LC mesophase and liquid–ODIC mesophase was proposed [20].

The high resolution of BDS measurements and resulting dielectric constant values enabled a subtle insight, shown in the inset in Figure 4 for crystal (Phase II). It also revealed a slight but detectable ‘jump’ for $\varepsilon \left(T\right)$ changes, ca. 10 K below the transition to the solid crystal phase, and the continuous change characterized by $d\varepsilon \left(T\right)/dT>0\;\;\leftarrow d\varepsilon \left(T\right)/dT<0$. Generally, such behavior is linked to the crossover, indicating parallel  ←antiparallel arrangement of dipole moments [46].

The pattern and values presented in Figure 4 correlate with the results reported by Tamarit et al. [40], which covered about 50% of the temperature range in ODIC phase of NPG. In the mentioned report, the Kirkwood–Frölich–Onsager model [46,47,53,54], generally developed for liquid dielectrics, was recalled to discuss the behavior in the ODIC phase. Their output relations do not enable portraying $\varepsilon \left(T\right)$ temperature evolution, offering a discussion of the isothermal, concentration-dependent behavior for a dipolar component dissolved in a non-dipolar solvent. Another possibility is the tests of the tendency toward dipole–dipole parallel or antiparallel arrangement via the Kirkwood factor, expressing the short-range dipole–dipole correlations [40,46,47,55]:

(6) $$g=\frac{9k_{B}T\varepsilon_{0}V_{m}\left(\varepsilon -\varepsilon_{\infty }\right)\left(2\varepsilon +\varepsilon_{\infty }\right)}{\varepsilon {\left(\varepsilon_{\infty }+2\right)}^{2}N_{A}\mu^{2}}$$

where $k_{B}$ is the Boltzmann constant, $\varepsilon_{0}=8.854\;\left(C^{2}J^{-1}m^{-1}\right)$ denotes vacuum electric permittivity, $V_{m}$ denotes the molar volume of the molecule, $\varepsilon_{\infty }$ is dielectric permittivity in the high-frequency limit, where the impact of the permanent dipole moment is absent, and μ is the permanent dipole moment.

Values of g>1 indicate the preference for the parallel and g<1 the antiparallel arrangement of neighboring dipole moments [40,46]. In [40], for NPG, the value g=1 was presented in the ODIC phase close to the transition to the crystal phase, and g=0.7 in the middle of the ODIC phase.

### 3.3. The Temperature Evolution of the Dielectric Constant and the Mossotti Catastrophe

**Figure 5:**
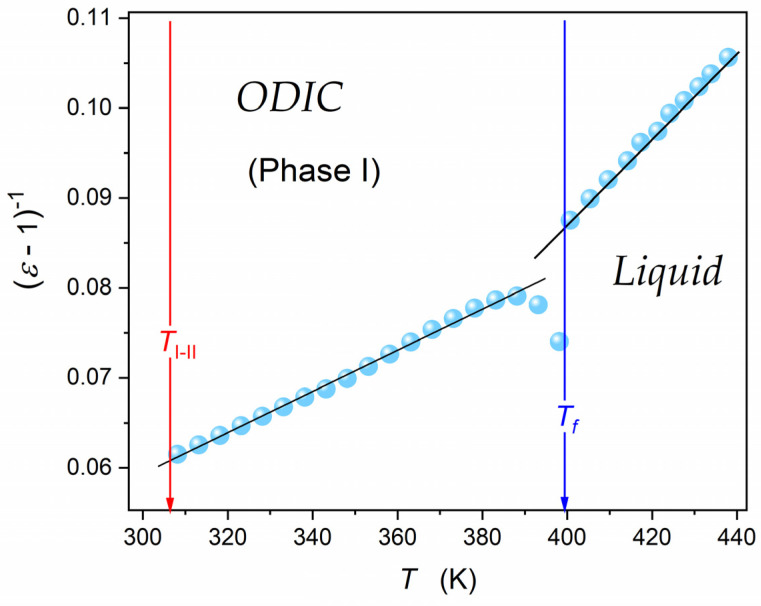
Temperature evolution of the reciprocal of dielectric susceptibility, linked to the dielectric constant via: χ=ε−1. The plot is based on $\varepsilon \left(T\right)$ data presented in Figure 4.

Figure 5 shows the re-analysis of data from Figure 4, presenting them as the reciprocal of dielectric susceptibility, χ=ε−1. It reveals a superior linear behavior in the liquid and ODIC phases, suggesting the following critical-like scaling pattern:

(7) $$\chi \left(T\right)=\varepsilon \left(T\right)-1=\frac{A}{T-T^{*}}\Rightarrow \chi^{-1}\left(T\right)=A^{-1}T-A^{-1}T^{*}$$

where $T^{*}$ is the singular, ‘critical’ temperature related to $\chi^{-1}\left(T^{*}\right)=0$, the amplitude A=const, and then $A^{-1}T^{*}=const$.

This result proves that the ‘linear’ behavior in Figure 4 is only virtual, appearing due to the ‘damping impact’ of the scale. A similar behavior was reported for another ODIC-forming material, cyclooctanol [20].

A similar temperature dependence as in Equation (7) appears for dielectric systems due to the Clausius–Mossotti local field model [44,45,51,52,54]. It considers the effective local field acting on a molecule within the dielectric by locating it in the center of a semi-macroscopic cavity surrounded by a homogeneous dielectric. Generally, it is described as follows [46,47,56]:

(8) $$F=E+E_{1}+E_{2}$$

where E is the external electric field, $E_{2}$ is the electric field created by elements/molecules close to the given molecule, and $E_{1}$ results from charges situated on the surface of the cavity.

For dielectric materials with a random distribution of elements/molecules (gases and liquids) or a regular crystalline lattice (solids), one can assume $E_{2}=0$, and the relatively simple consideration for the remaining contribution yields:

(9) $$E_{1}=\frac{P}{3\varepsilon_{0}}$$

where *P* is for the polarization and $\varepsilon_{0}=8.854\;pFm^{-1}$ denotes the vacuum electric permittivity.

Consequently, for the local electric field [46,56], we can obtain:

(10) $$F=E+\frac{P}{3\varepsilon_{0}}=E+\frac{\chi \varepsilon_{0}E}{3\varepsilon_{0}}=E\left(1+\frac{\chi }{3}\right)=E\left(1+\frac{\chi }{3}\right)=E\frac{\chi +3}{3}=\chi \varepsilon_{0}E/N\alpha$$

where the basic relation $P=\chi \varepsilon_{0}E=N\alpha F\;\;\Rightarrow F=\chi \varepsilon_{0}E/N\alpha$ was taken into account.

The above relation can be considered in two equivalent forms, taking into account that the number of polarizable molecules in a unit volume $N=N_{A}\rho /M$, where M denotes the molar mass and *ρ* is for the density [44,54]:

(11) $$II=\frac{N_{A}\alpha_{P}}{3\varepsilon_{0}}=\frac{M}{\rho }\frac{\chi }{\chi +3}$$

(12) $$\chi^{\prime }=\frac{P}{\varepsilon_{o}E}=\frac{N\alpha_{P}/\varepsilon_{0}}{1-N\alpha_{P}/{3\varepsilon }_{0}}$$

Equation (11) defines the molar polarizability, *II*, or the molar refraction, R, for light-related frequencies, where the Maxwell dependence obeys: $\varepsilon =1+\chi =n^{2}$, where n denotes the refractive index. Values of *II* and R are significant practical tools in chemical physics applications.

von Hippel [56] noted that for dipolar dielectrics, the contribution to the dielectric constant from electronic $\left(\alpha_{e}\right)$ and atomic $\left(\alpha_{a}\right)$ polarizations, expressed via $\varepsilon_{\infty }$, is minimal in comparison to the impact of permanent dipole moments. He accepted Debye’s estimation for the latter, which yielded $\alpha_{P}=\left(\alpha_{a}+\alpha_{e}\right)+\alpha_{dip}=\alpha_{ind.}+\mu^{2}/3k_{B}T\approx \mu^{2}/3k_{B}T$, and after the substitution to Equation (12), the following relation was obtained [56]:

(13) $$\chi =\varepsilon -1=\frac{3T_{C}}{T-T_{C}}$$

where $T_{C}=N\mu^{2}/9\varepsilon_{0}k_{B}$ and $N=N_{A}\rho /M$.

This is the famous ‘Mossotti Catastrophe’, suggesting that in an arbitrary system composed of permanent dipole moments, non-interacting or weakly interacting, a singularity resembling the Weiss-type pre-transitional effect, known for the paraelectric phase in the way toward the ferroelectric state, appears. von Hippel presented a famous example of water, where he estimated $T_{C}\approx 1520\;K$, which suggests a ferroelectric state for lower temperatures. Finally, von Hippel concluded [56]: ‘*Hence water should solidify by spontaneous polarization at high temperature, making life impossible on this earth*!’. This picturesque example has often been cited in subsequent decades to illustrate the consequences of exceeding a model’s assumptions, leading to paradox predictions, absent in nature. To avoid this paradox, Onsager developed the model in [57], explicitly considering the Debye concept, considering short-range interactions by a different cavity definition, and introducing the reaction field associated with the feedback interaction between the cavity and the permanent dipole moment. This approach was further developed by Kirkwood, Frölich, and followers, leading to the agreement with experiments in real dielectric liquids [46,47,53,54,55,56,57]. The problem, however, constitutes limited possibilities for describing temperature changes. In analyzing experimental data, the Kirkwood factor discussion plays a leading role (Equation (6)). Consequently, a ‘paradigm’ emerged that the Clausius–Mossotti model obeys only for a gaseous or non-dipolar liquid dielectric [46,47,53,54,55,56,57,58,59,60,61,62]. However, there are numerous solid-state systems where this model is widely accepted, e.g., in solid or liquid crystalline ferroelectric materials or relaxor ceramics [60,61,62,63,64,65,66,67,68]. This issue is resumed and developed in the authors’ recent report (ADR and SJR) [69].

It should be noted that von Hippel [56] overlooked a primary problem. Namely, he assumed $\rho =1\;g{cm}^{-3}$ for the density of water, also at the extreme temperatures T>1500 K. This is possible only under multi-GPa pressure, which may lead to the appearance of exotic states of matter.

Results presented in Figure 5 for NPG and the recent evidence for cyclooctanol [20] explicitly show that dipolar liquid or quasi-liquid systems with the Mossotti Catastrophe behavior exist. This can be considered as the cancellation of von Hippel’s ‘catastrophic paradigm’, which significantly changes some basics of Dielectrics Physics, such as the canonic picture presented in classic monographs [46,47,53,54,55,56].

### 3.4. The Link to the Pre-Transitional, Fluctuation-Related Behavior

In [20], it was indicated that one can consider the appearance of pre-transitional fluctuations both in the liquid and the ODIC mesophase, namely, with the ODIC-like order and the ‘chaotic surrounding’ in the liquid phase and in the ODIC phase related to the ‘frozen’ orientational arrangement within the orientationally free quasi-liquid mesophase surrounding. It led to the following relation for ‘static’ dielectric susceptibility [20,70]:

(14) $$\chi \left(T\right)=\varepsilon \left(T\right)-1\propto {\langle \Delta M^{2}\rangle }_{V}\chi_{T}=\frac{\chi_{0}{\langle \Delta M^{2}\rangle }_{V}}{T-T^{*}}$$

where $\chi_{T}$ is the compressibility, in the given case, meaning susceptibility related to the coupled-order parameter, and ${\langle \Delta M^{2}\rangle }_{V}$ is the mean square of the order parameter fluctuations, i.e., the metric of fluctuations of the local order parameter, which is related to the difference in dielectric constants between fluctuations recalling features of the next phase and the surrounding in the specified type of materials.

For the the liquid phase and the adjacent ODIC mesophase, one can assume ${\langle \Delta M^{2}\rangle }_{V}=const$, which directly yields the same temperature dependence as Equation (7), in agreement with Figure 5. The model implemented in [20] recalls the authors’ analysis of nonlinear dielectric properties on approaching the critical consolute point and the isotropic–nematic transition in LC materials [70].

One can conclude that for dipolar ODIC-forming materials, both in the liquid and quasi-solid mesophase, the Clausius–Mossotti local field model, and its crucial output, the Mossotti Catastrophe (Equation (13)) can be obeyed. This is due to the possibility of the free orientation of permanent-moment dipoles and the practical lack of short-range interactions associated with their translational ‘localization’. This means that the canonic condition of the Clausius–Mossotti local field model is fulfilled.

Considering the results of the current report and that presented in [20,70], the need arises for an exceptional universal model description linking ODIC-forming materials in the liquid and ODIC phases, liquid crystalline materials in the isotropic liquid phase, and maybe the homogeneous phase of critical binary mixtures, particularly under the strong electric field inducing the uniaxial anisotropy.

### 3.5. The Evolution of Electric Conductivity and DC Electric Conductivity

DC electric conductivity is the metric of the ability to conduct a direct, in-phase electric current in a specific material. Heuristically, DC conductivity can be treated as a dynamic equivalent of the dielectric constant since it is also a frequency-independent quantity over a wide (low and static) frequency range. This is shown in Figure 3, where the horizontal domain for the frequency dependence of the imaginary part of electric conductivity, DC electric conductivity, $\sigma_{DC}=\sigma \approx const$. Such behavior appears only in the liquid and ODIC phases, and it is absent in the solid crystal phase.

**Figure 6:**
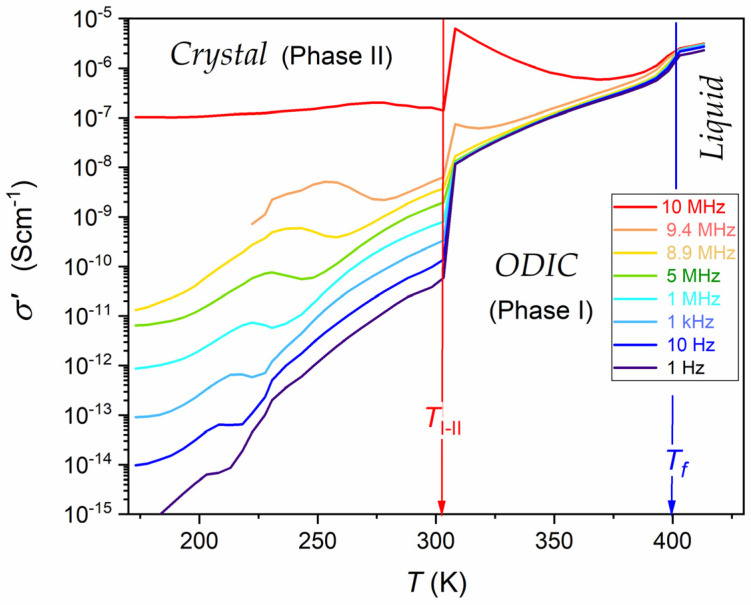
Temperature dependence of the real part of electric conductivity for a set of frequencies indicated in the plot. Arrows show subsequent phase transitions. Results are presented as curves linking experimental data points—to support the view. Note the overlapping of $\sigma \left(T\right)$ dependencies for a set of frequencies in the liquid and ODIC phases. It disappears for high frequencies due to the rising impact of the dielectric relaxation process. The mentioned overlapping is absent in the solid crystal phase, reflecting the lack of DC electric conductivity for this phase.

Figure 6 shows temperature dependencies of electric conductivity for a set of frequencies. Notable is the overlapping in the liquid and ODIC phases for less than 5 MHz, which agrees with the frequency domain of DC electric conductivity in Figure 3, as discussed. Rising distortions appear for higher frequencies, which can be considered the impact of relaxation processes. The split in the crystal phase confirms the lack of canonic DC electric conductivity in this region.

**Figure 7:**
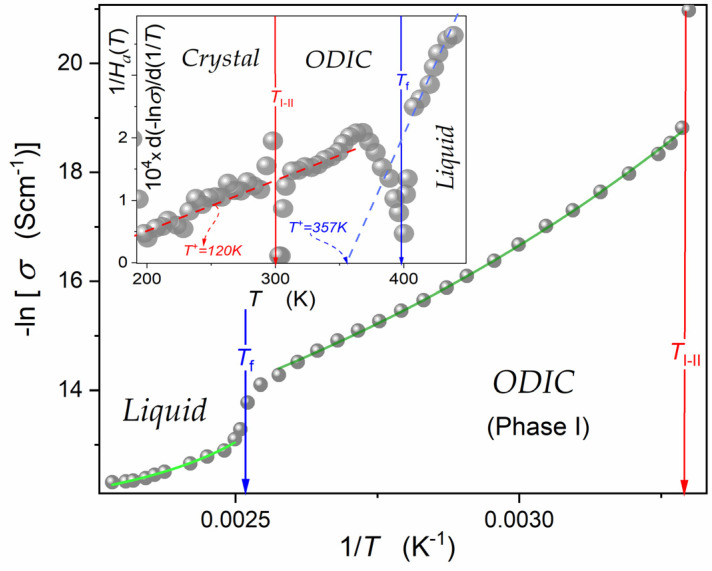
Temperature dependence of the DC electric conductivity reciprocal in the liquid and ODIC phases of NPG, using the Arrhenius scale. The inset shows the evolution of the apparent activation enthalpy reciprocal, as defined in the plot and Equation (14). The curves following experimental data in the central part of the plot are related to Equation (15) with the following parameters: $C_{\Gamma }=0.19\times 10^{-4}(S{cm}^{-1})$, $T_{C}=120\;K$, and the exponent Γ=3.40 (green curve, ODIC phase), and $C_{\Gamma }=0.18\times 10^{-4}\;(S{cm}^{-1})$, $T_{C}=357\;K$, and the exponent Γ=0.63 (light-green curve, liquid phase).

As mentioned in the Introduction Section, in [43], the VFT portrayal was suggested for the DC electric conductivity in all phases of NPG, namely, for the liquid phase in the tested range $\Delta T_{liq.}=10\;K$, for the ODIC ‘mesophase’ in the tested range $\Delta T_{ODIC}=59\;K$, and for the crystal phase in the tested range $\Delta T_{Cryst.}=17\;K$. The VFT portrayal was validated by the linear dependence for the so-called ‘Stickel plot’ ${\left(dln\sigma_{DC}/dT\right)}^{-1/2}$ vs. *T*, originally developed to test pre-glassy changes in the primary relaxation time [71,72,73]. Figure 7 presents the temperature dependence of DC electric conductivity in the liquid and ODIC phases based on data presented in Figure 6. Results are presented using the Arrhenius-type scale, $ln\sigma^{-1}$ vs. 1/T, i.e., the standard representation for dynamic processes, at which the basic Arrhenius pattern with constant activation energy manifests via a linear dependence. There is no such behavior in Figure 7.

The inset in Figure 7 presents the temperature evolution of the reciprocal of apparent activation enthalpy, $H_{a}\left(T\right)$, proportional to the so-called steepness index. Notable is the link to the ‘technical Stickel plot’ [71,72,73,74,75,76]. It covers all NPG phases tested in the given report. The emerging linear behavior validates the following dependence [75,76]:

(15) $${H_{a}\left(T\right)=\left[\frac{dln\sigma^{-1}\left(T\right)}{d\left(1/T\right)}\right]}^{-1}=HT+HT^{+}\frac{dln\sigma^{-1}\left(T\right)}{d\left(1/T\right)}=\frac{H}{T-T^{+}}$$

where H=const, and $T^{+}$ is the extrapolated singular temperature related to the ${\left[H_{a}\left(T^{+}\right)\right]}^{-1}=0$, $HT^{+}=const$ condition.

Such ‘universal’ behavior was first noted in [76] for the so-called apparent fragility of the primary relaxation time, which is proportional to the related apparent activation enthalpy or, equivalently, the steepness index. It directly leads to the ‘activated and critical’ equation formulated by Aleksandra Drozd-Rzoska [76]. For the DC electric conductivity considered in this report, it has the form:

(16) $$\sigma^{-1}\left(T\right)=C_{\Gamma }{\left(\frac{T-T^{+}}{T}\right)}^{-\Gamma }{\left[\exp\left(\frac{T-T^{+}}{T}\right)\right]}^{\Gamma }=C_{\Gamma }{\left(t^{-1}\exp t\right)}^{\Gamma }$$

where $t=\left(T-T^{+}\right)/T$, $T^{+}$ is the extrapolated singular temperature, the pre-factor $C_{\Gamma }=const$, and the exponent Γ=const.

The parameterizations of experimental data in Figure 7 are related to Equation (16). They are supported by parameters obtained by the analysis presented in the inset in Figure 7, in agreement with Equation (15). It covers the liquid phase in the range $\Delta T_{liq.}\approx 30\;K$, and $\Delta T_{ODIC}\approx 85\;K$ in the ODIC phase.

It is worth stressing that the authors’ recent work explicitly evidenced that the VFT relation is fundamentally justified only for a limited number of systems exhibiting ‘glassy’ dynamics, and this group does not include ODIC-forming materials [75].

### 3.6. Low-Frequency Behavior of Dielectric Permittivity and the Loss Factor

Temperature dependencies of the real and imaginary parts of dielectric permittivity in the low-frequency domain remain a puzzling issue, if not a cognitive gap [43,46,47,56].

For the imaginary part, $\varepsilon ”\left(f,T\right)$, it can be considered using the DC electric conductivity behavior discussed above. Namely, $\varepsilon^{\prime \prime }\left(f,T\right)=\sigma_{DC}\left(T\right)/\omega \varepsilon_{0}$, where ω=2πf, and for $\sigma_{DC}\left(T\right)$ behavior, one can use the parameterization by Equation (16). For the real part of dielectric permittivity, in some reports, the dependence $\varepsilon^{\prime }\left(f\right)\propto f^{-p}$, i.e., ${log}_{10}\varepsilon^{\prime }\left(f\right)\propto {-plog}_{10}f$ ([75] and references therein) was suggested. However, it poorly correlated with the experimental evidence, as visible in Figure 1 and discussed in [77].

**Figure 8:**
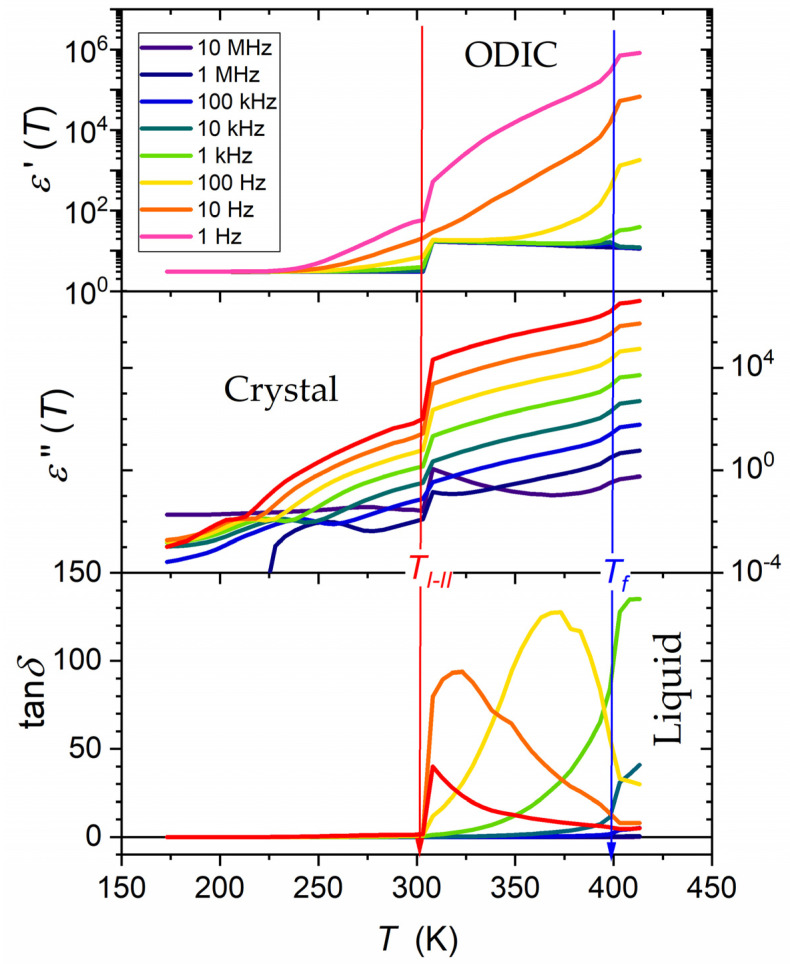
Temperature evolutions of the real ($\varepsilon^{\prime }\left(f\right)$) and imaginary ($\varepsilon^{\prime }\prime \left(f\right)$) parts of dielectric permittivity and for the dissipation factor, $D\left(f\right)=tan\delta \left(f\right)=\varepsilon^{\prime }\prime \left(f\right)/\varepsilon^{\prime }\left(f\right)$, for selected frequencies in tested phases of NPG. Arrows indicate phase transition. $\varepsilon^{\prime }\left(f\right)$) and $\varepsilon^{\prime }\prime \left(f\right)$ are in the semi-log scale, and $tan\delta \left(f\right)$ is exclusively in the linear scale. Results are presented as curves linking experimental data points to support the view. Note the strong pre-melting/postfreezing-type effects on the solid crystal side of the strongly discontinuous phase transition. These effects disappeared for the evolution of the energy dissipation factor.

Figure 8 present $\varepsilon^{\prime }\left(f,T\right)$ and $\varepsilon^{\prime \prime }\left(f,T\right)$ temperature evolutions in NPG for selected frequencies covering the static and the LF domains. For $\varepsilon^{\prime }\left(f,T\right)$, experimental data overlapped for frequencies in the static domain for the liquid and ODIC phases. In the LF domain, a ‘fan’ of temperature dependencies appeared. For $\varepsilon^{\prime }\prime \left(f=const,T\right)$ dependencies, the overlapping of data for different frequencies using the definition of DC electric conductivity was possible: $\varepsilon^{\prime \prime }\left(f,T\right)\omega =\sigma_{DC}\left(T\right)/\varepsilon_{0}$.

Figure 8 focuses also on the temperature evolution of the magnitudes linking the above contributions, namely, the dissipation factor, $D=\tan\delta \left(f,T\right)=\varepsilon^{\prime \prime }\left(f,T\right)/\varepsilon^{\prime }\left(f,T\right)$. This magnitude is commonly used in engineering applications but scarcely in fundamental analysis of dielectric materials. It estimates the power loss under the action of the external electric field, which can be converted into heat, namely [47,78,79,80,81,82,83]:

(17) $$P=Qtan\delta =\omega CV^{2}tan\delta =\varepsilon_{0}\varepsilon^{\prime \prime }E^{2}$$

It is often discussed via the quality factor, Q=1/D. It is a significant metric of the dielectric materials’ property commonly referred to in engineering applications. However, it is hardly considered in fundamental studies. In this context, it is worth recalling that:

(18) $$\varepsilon^{*}=\varepsilon^{\prime }-i\varepsilon^{\prime \prime }=\varepsilon^{\prime }\left(1-i\times tan\delta \right)\;\mathrm{and}\;tan\delta =\frac{\varepsilon^{\prime \prime }}{\varepsilon^{\prime }}$$

Figure 8 shows the temperature dependences of the dissipation factor, $D\left(T\right)$, using data presented above. It is notable that $\varepsilon^{\prime }\left(T\right)$ and $\varepsilon^{\prime \prime }\left(T\right)$ evolutions were presented using the semi-log scale since their values changed by almost six decades for the discussed frequencies. This range was qualitatively reduced when considering the ratio of $\varepsilon^{\prime }\left(T\right)$ and $\varepsilon^{\prime \prime }\left(T\right)$, so the linear scale could be informative for the dissipation factor.

The characteristic feature was the dissipation maximum in the ODIC phase, for the lowest tested frequency occurring near the ODIC−Crystal transition and shifting toward the Liquid−ODIC transition when the frequency increased. For frequencies larger than ~5 kHz, only a dissipation decay in the ODIC mesophase upon cooling occurred. The dissipation was negligible in the Crystal phase. Negligible dissipation also appeared in the ODIC and liquid phases for f>100 kHz.

**Figure 9:**
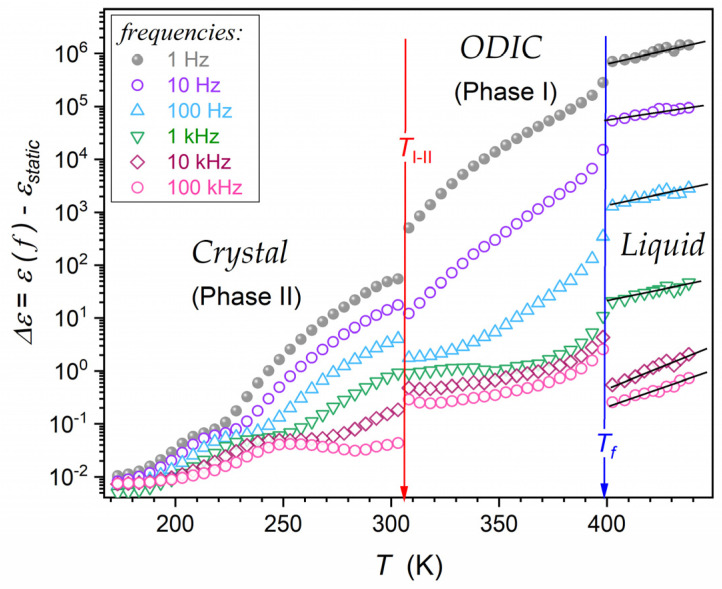
The temperature behavior of the low-frequency (LF) contribution of the real part of dielectric permittivity in NPG, presented via the semi-log scale. Arrows indicate phase transitions. Linear changes in the liquid phase validate exponential changes (Equation (19)). The plot is based on data presented in Figure 6, using the frequency f=5 MHz as the reference for determining the dielectric constant, namely, $\varepsilon^{\prime }\left(f=5\;MHz\right)=\varepsilon_{static}=\varepsilon$.

**Figure 10:**
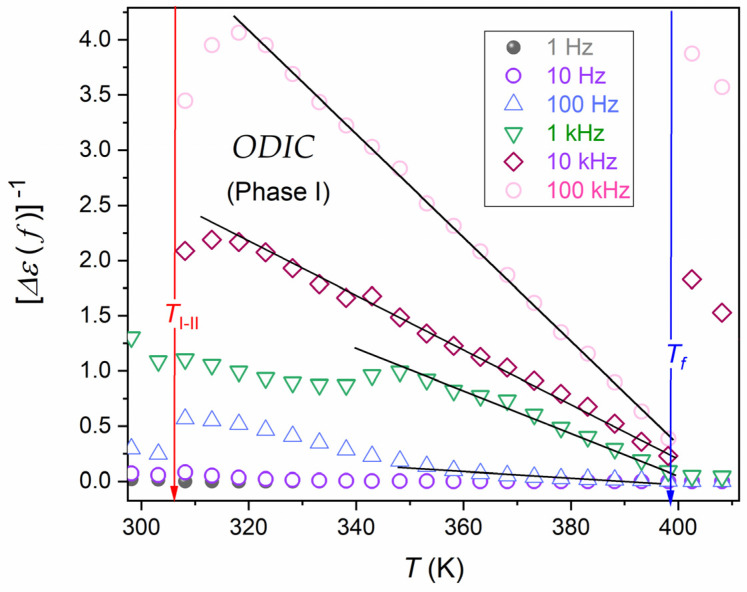
The temperature behavior of the reciprocal of the low-frequency (LF) contribution to the real part of dielectric permittivity in the ODIC phase of NPG. Arrows indicate phase transition. Linear changes in the liquid phase validate the behavior outlined by Equation (20). The plot is based on data presented in Figure 2, using the frequency f=5 MHz as the reference for determining the dielectric constant, namely, $\varepsilon^{\prime }\left(f=5\;MHz\right)=\varepsilon_{static}=\varepsilon$.

The issue of a reliable scaling relation, which can describe the behavior of the real part of dielectric permittivity, remains a challenge, which constitutes a fundamental cognitive gap and creates a problem because of the significance of this domain in applications [45,84,85,86,87,88,89,90,91]. Figure 9 and Figure 10 are related to a possible parameterization of the real part of dielectric permittivity, focusing solely on the LF contribution. It is realized by testing the magnitude, $\Delta \varepsilon^{\prime }\left(f,T\right)=\varepsilon^{\prime }\left(f,T\right)-\varepsilon \left(T\right)$, obtained by subtracting the static part, $\varepsilon^{\prime }=\varepsilon \left(T\right)$, from the total value, $\varepsilon^{\prime }\left(f,T\right).$ Figure 9 presents $\Delta \varepsilon^{\prime }\left(f,T\right)$ changes in the semi-log scale, revealing a linear pattern. This linear dependence appearing in the liquid phase indicated the following temperature parameterization:

(19) $$\Delta \varepsilon^{\prime }\left(f,T\right)=\Delta_{0}^{f}exp\left(F\times T\right)$$

where $\Delta_{0}^{f}$ is the pre-factor, related to the specified frequency, f, and amplitude F=const.

Such behavior was recently noted in the isotropic liquid phase of liquid crystalline nematogenic 4-methoxybenzylidene-4’–butylaniline (MBBA) [77].

Figure 10 shows that such parametrization was absent in the ODIC mesophase. However, the presentation of the same data using the following scaling: ${\left[\Delta \varepsilon \left(f,T\right)\right]}^{-1}$ vs. *T*, revealed explicit linear changes. It led to the following parameterization:

(20) $${\left[\Delta \varepsilon \left(f,T\right)\right]}^{-1}=a-bT\Rightarrow \Delta \varepsilon \left(f,T\right)=\varepsilon^{\prime }\left(f,T\right)-\varepsilon \left(T\right)=\frac{1}{a-bT}=\frac{1/b}{a/b-T}=\frac{1/b}{T^{f}-T}$$

where $T^{f}$ is the singular temperature obtained from the extrapolation of the emerging temperature dependence in the ODIC phase via the condition ${\left[\Delta \varepsilon \left(f,T^{f}\right)\right]}^{-1}=0$.

## 4. Conclusions

The report discussed the low-frequency, static, and dynamic dielectric properties of neopentyl glycol, an ODIC-forming material of growing importance in applications that still requires fundamental insight support. The nature of ODIC-forming systems indicates that dielectric studies are essential for explaining and modeling their properties.

The basic dielectric property is the dielectric constant, related to the static frequency domain. It was shown that its evolution in the liquid and ODIC phases followed a pattern reminiscent of the Mossotti Catastrophe pattern, which follows directly from the Clausius–Mossotti local field concept. Formally, it is ‘forbidden’ for dipolar liquid dielectrics. From this report on neopentyl glycol and the recent study [20] on cyclooctanol, such a description is possible for ODIC-forming, dipolar dielectric materials. It obeys both the liquid and ODIC phases.

The dielectric constant is related to the real part of dielectric permittivity. DC electric conductivity can be considered its specific equivalent for dynamic properties and related to the imaginary part of dielectric permittivity. This work showed critical-like changes in apparent activation enthalpy associated with electric conductivity. This characterization led directly to the description of changes in electrical conductivity using the critical and activated Drozd-Rzoska dependence (Equation (16)) [77]. The work also attempted to search for patterns of scaling temperature changes for changes in electrical conductivity in the low-frequency area. The description of extreme changes in the ε′(f,T) value remains a challenge for dielectric physics. This work showed the emergence of two simple and well-defined scaling relations in the ODIC and liquid phases. In the latter case, it was consistent with that recently reported for the isotropic liquid phase of a nematogenic liquid crystalline material. This report for the ODIC mesophase and recent work on nematic mesophases [20] also showed highly characteristic, pre-transitional-like changes in the dissipation factor, a quantity combining the real and imaginary parts of the dielectric permittivity. We emphasized this issue. Finally, the authors would like to indicate the relatively small step changes in the dielectric constant and tgδ for the phase transition from the isotropic liquid to the ODIC phase, in comparison to ‘huge’ step changes for the phase transition from the ODIC phase to the crystalline phase. Such a mutual relationship is consistent with Timmermans ‘classic’ observation [9], suggesting a weak and strong sequence of phase transitions based on specific heat studies.

For ODIC-forming materials, dielectric studies are essential due to their association with translational freezing and freely rotating molecules and coupled permanent dipole moments. This report showed that the picture emerging from dielectric studies in NPG, which should be considered a specific representative of ODIC-forming materials, differs from what has been suggested so far. It showed virtually exotic features, such as Mossotti Catastrophe-type behavior. The pre-melting/postfreezing-type effects on the solid crystal side of the discontinuous transition or the possible link between the behavior in the liquid and ODIC mesophase transition is worth stressing. All these results can suggest opening a new multitude of cognitive pathways for further studies.

## Data Availability

Experimental data are available from the authors upon reasonable request.
